# Public Attitudes toward the Final Disposal of Radioactively Contaminated Soil Resulting from the Fukushima Daiichi Nuclear Power Station Accident

**DOI:** 10.1007/s00267-024-01938-w

**Published:** 2024-02-02

**Authors:** Momo Takada, Michio Murakami, Susumu Ohnuma, Yukihide Shibata, Tetsuo Yasutaka

**Affiliations:** 1 Institute for Geo-Resources and Environment, National Institute of Advanced Industrial Science and Technology, Tsukuba, Ibaraki Japan; 2 Center for Infectious Disease Education and Research, Osaka University, Suita, Osaka Japan; 3 Department of Behavioral Science, Faculty of Humanity and Human Sciences / Center for Experimental Research in Social Sciences, Hokkaido University, Sapporo, Japan; 4 Faculty of Humanity and Human Sciences, Hokkaido University, Sapporo, Japan

**Keywords:** Risk perception, Inequality, Protected values, Radiocesium, Moral foundation, Procedural fairness

## Abstract

Radioactively contaminated soil from the Fukushima Daiichi nuclear power station accident in 2011 is required by law to be finally disposed of outside Fukushima Prefecture by 2045. To gain public acceptance of this policy, it is essential to promote understanding and nationwide discussion. We conducted a web-based survey of 2000 people in Japan to examine public attitudes toward final disposal of the contaminated soil outside Fukushima Prefecture. Results show that policy approval was negatively correlated with perceived risk of a final disposal site, sense of inequity associated with building a final disposal site near residential areas, and values that are absolutely non-negotiable or protected from trade-offs with other values (protected values). Policy approval was positively correlated with high levels of interest in the Fukushima accident and subjective knowledge of decontamination and the policy. Respondents’ comments and opinions about the policy indicated that respondents who approved of the policy accepted burden sharing, while those who disapproved were unconvinced by the rationale behind disposal outside Fukushima Prefecture and were dissatisfied by the lack of information disclosure and transparency. While the government’s efforts to disseminate information about the current status and future of Fukushima have been effective to a certain extent, they are insufficient to achieve widespread public understanding of the policy. Our results indicate that attention needs to be paid to procedural fairness and explanations of risks.

## Introduction

Many studies have been conducted on public acceptance of NIMBY (Not in My Back Yard) facilities, which are facilities that are recognized as necessary but undesirable to the surrounding community. These include municipal, industrial, radioactive, and nuclear facilities. For example, studies have shown that a perception of high risks associated with the facility negatively impacts facility acceptability (Slovic 1987; Slovic et al. 1991; Sjöberg 2004; Shirai et al. 2023). The burden of such a facility is unevenly distributed in a region and can lead to a sense of “why is this in our own region when it could be in another region?” (Willard and Swenson 1984), which has a negative impact on facility acceptance (Yokoyama et al. 2021, Takada et al. 2022). Protected values, which are values that are absolutely non-negotiable or are protected from trade-offs with other values (Baron and Spranca 1997), also prevent acceptance (Krütli et al. 2012). For example, some people may consider the risks associated with such a facility to be unacceptable, which cannot be traded off with any benefits. Protected values have been reported to be positively correlated with risk perception and inequity (Yokoyama et al. 2021). On the contrary, transparency and openness in decision making, as well as opportunities for public input, increase acceptance (Coninck et al. 1999; Nonami et al. 2015; Yokoyama and Ohnuma 2018).

The Fukushima Daiichi nuclear power station accident in Japan in 2011 (hereafter Fukushima accident) generated contaminated soil and combustible materials containing radiocesium (hereafter contaminated soil), and site selection for the final disposal of the contaminated soil remains an issue. Between the accident and March 2018, approximately 13 million m^3^ of contaminated soil was generated from decontamination activities conducted in residential areas, farmland, forests near residential areas, and roads in Fukushima Prefecture (Ministry of the Environment 2023a). Approximately 80% of the contaminated soil has a radioactivity of less than 8000 Bq/kg, and is recyclable as soil material according to regulations (Ministry of the Environment 2023b). Therefore, the contaminated soil is different from general radioactive waste from nuclear facilities and can be disposed of as non-radioactive waste (IAEA 2011). The contaminated soil has been stored in an interim storage facility of approximately 1600 hectares located near the Fukushima Daiichi nuclear power station. In line with the Japan Environmental Storage & Safety Corporation Law (Law No. 44 of 2003, promulgated on May 16, 2003), the government has decided that the contaminated soil will be transported out of Fukushima Prefecture for final disposal by 2045, and at the same time the soil will be recycled and used as construction materials throughout Japan. The 2045 deadline is based on the legal provision that requires final disposal to be completed no later than 30 years after the start of operation of the interim storage facility, and was set in consideration of the half-life of ^137^Cs, which is the main contaminant (House of Representatives 2014). According to the government, the decision to implement final disposal outside Fukushima Prefecture (hereafter final disposal policy) is “a result of a comprehensive judgment based on the fact that residents in Fukushima Prefecture are already bearing an excessive burden” (House of Representatives 2014), which was approved by the Cabinet in 2011. Currently, technical development for final disposal is underway, but candidate sites have not yet been identified. The Ministry of the Environment intends to increase nationwide public understanding of the developments toward final disposal (Ministry of the Environment 2023c). It has a special website dedicated to the dissemination of information on the final disposal and recycling of the contaminated soil outside Fukushima Prefecture (Ministry of the Environment 2023d). It has also organized tours to inform people about environmental restoration efforts in Fukushima and held interactive meetings to present their efforts in preparation for the recycling of the contaminated soil (Ministry of the Environment 2023d). In general, waste disposal is more difficult if it is not carried out at the site of generation (Johnson 1987). Moreover, with the lapse of time, public interest in the Fukushima accident is likely to wane. Accordingly, social aspects such as consensus building rather than technical aspects are expected to be the difficulties that need to be negotiated to implement and complete the final disposal policy by 2045.

Following the Fukushima accident, studies have been conducted on public acceptance of the disposal of low radioactive materials, such as contaminated soil from environmental remediation sites. Takada et al. (2022) reported that questionnaire respondents chose to locate a final disposal site for the contaminated soil in Fukushima far from residential areas, and that they attached importance to distributive and procedural fairness. Shirai et al. (2023) used structural equation modeling to show that risk perception decreases acceptability, social benefits increase acceptability, and trust and intergenerational expectations are important factors affecting the acceptability of the disposal; the impact of each of these factors on acceptability varies depending on the level of emphasis placed on moral norms, and moral foundations have a strong relationship with policy discussion (Graham et al. 2011). Although these findings have much in common with the studies on NIMBYs mentioned above, the characteristics of contaminated soil differ from those of conventional waste disposal from a social perspective (Takada et al. 2022): for example, in the case of contaminated soil, the source region is not a potential candidate for the final disposal site. However, most of these previous studies examined acceptability under the assumption that a candidate disposal site has been decided, and few studies have examined acceptability of the final disposal policy prior to site selection. As mentioned above, the final disposal of the contaminated soil from the Fukushima accident will be at site(s) outside Fukushima Prefecture, and therefore it will be important to include the general public throughout Japan as stakeholders and understand their attitudes toward the final disposal policy before site selection.

This study examines public attitudes in Japan toward the final disposal of the contaminated soil outside Fukushima Prefecture and related factors through a web-based survey. Our research questions are as follows. (1) How many people in Japan know and agree with the policy of transporting the contaminated soil out of Fukushima Prefecture for final disposal? (2) While risk perception, sense of inequity, protected values, interest in the Fukushima accident, and subjective knowledge levels of decontamination and the policy affect acceptability of NIMBY facilities at candidate sites, are these factors also related to public attitudes toward the final disposal policy? (3) Do the factors that affect acceptability differ among groups with different levels of emphasis on moral norms? Finally, through text analysis of comments and opinions about the policy, we assess (4) whether respondents’ focus varies with attitudes toward the final disposal policy. We analyzed the impacts of the benefits that respondents had received from Tokyo Electric Power Company (TEPCO), and respondents’ interests, knowledge, and sense of involvement with the Fukushima accident on their agreement with the final disposal policy. We compared public attitudes from two regions that are comparable in terms of population and size of economy, but which differ in terms of residents’ relationship with TEPCO, interests, knowledge, and sense of involvement. Residents in Kanto region had benefited from the electricity generated by the Fukushima nuclear power station, while residents in Kansai region, which is at a considerable distance from Fukushima, had not. The two regions are comparable in terms of population and economy. Other regions in Japan could also be potential sites for final disposal but they differ considerably from Kanto region in terms of population and economy. Therefore, in this study, we focused on Kanto region as a beneficiary of the Fukushima nuclear power station and Kansai region as a control group. Our study can contribute to solving the problem of radioactive waste disposal generated by environmental decontamination after the Fukushima accident, and to preparing for possible future large-scale nuclear accidents. This study is also useful to inform similar NIMBY facility siting efforts in which there are a wide range of candidate sites with no geographical constraints and it is necessary to consider the entire population as stakeholders and understand their attitudes before deciding on a candidate site.

## Materials and Methods

### Samples and Data Collection

The study was approved by the research ethics committee of Hokkaido University (receipt number, FY2022-22). The respondents were between 20 and 69 years old and lived in Kanto region (eight prefectures located approximately 60–350 km from the Fukushima Daiichi nuclear power station: Tokyo, Kanagawa, Saitama, Chiba, Tochigi, Gunma, Ibaraki, and Yamanashi) and Kansai region (five prefectures located 500–700 km from the Fukushima Daiichi nuclear power plant: Shiga, Kyoto, Osaka, Nara and Wakayama). Fukushima Prefecture was not included in this study. Kanto region was chosen because its electricity is supplied by TEPCO and it is considered to be a beneficiary of the Fukushima Daiichi nuclear power station. After the accident, planned power outage was immediately implemented in Kanto region because of the reduced power supply capacity of TEPCO (Ministry of Economy, Trade and Industry 2011). Kansai region was chosen for comparison with Kanto region based on our previous online survey of Japanese citizens living outside Fukushima Prefecture and their acceptance of the final disposal of radioactively contaminated soils (Takada et al. 2022; Shirai et al. 2023). Results of our earlier survey confirmed that interest and subjective knowledge of this final disposal policy differed among regions. Interest in Kansai region (3.0 with 1 = not interested, 5 = very interested) was lower than that in Kanto (3.2) region and comparable to that in Chugoku (3.0), Shikoku (3.1), and Kyushu and Okinawa (3.0) regions. Interest levels were 3.2 in Hokkaido and Chubu and 3.3 in Tohoku (excluding Fukushima). Subjective knowledge levels were 1.8 (1 = never heard, 4 = know well) in Tohoku (excluding Fukushima) and Chubu regions, 1.7 in Kanto, Kansai, Hokkaido, Chugoku, and Shikoku regions, and 1.6 in Kyushu and Okinawa regions. Kansai region was also chosen for comparison with Kanto region because it is far enough away from Fukushima and not a beneficiary of the Fukushima Daiichi nuclear power station. Unlike Kanto region, Kansai region did not experience the electricity crisis caused by the accident, and residents’ sense of involvement in the accident is expected to be lower than that in Kanto region. Kansai region has the second largest economy after Kanto region in Japan and it corresponds to the area supplied by Kansai Electric Power Company, which also has nuclear power stations.

Respondents registered with the online survey company (Cross Marketing Inc.) through which data were collected. The web survey was conducted in December 2022 and received 2000 responses. There were 1000 responses from each of the regions (the survey continued until 1000 responses were received from each region). The number of respondents meets the requirement for representative sample size for a 5% margin of error and a 99% confidence interval (approximately 700 persons from each region), according to Serdar et al. (2021). Respondents were evenly distributed in terms of age group (20s, 30s, 40s, 50s, and 60s) and gender (near equal numbers of female and male respondents based on the four response options—see below). Respondents were incentivized with web points, which could be used to purchase items online, and were worth approximately 20 Japanese Yen (US$0.17).

All respondents participated voluntarily and gave consent for the use and publication of their responses for research purposes. They were first provided with detailed information, including an overview of the survey and the intended use of the data, via text. Only those who selected the option of agreeing to participate in the survey continued to the next phase. They were then asked to provide basic information (age, gender—no answer/other/female/male, and prefecture) and read a preliminary explanation of the final disposal for at least 30 s. The preliminary explanation described the environmental contamination caused by the Fukushima accident, the amount of contaminated soil and the amount of money spent on decontamination, the current situation at the interim storage facility, the law on final disposal and its cabinet decision (“as a result of a comprehensive judgment based on the fact that residents in Fukushima Prefecture are already bearing an excessive burden”), and that the specific details of the final disposal have not yet been decided (Online Resource 1).

### Questionnaire

Twelve questions were used to collect data on seven factors: interest, subjective knowledge of decontamination and the policy, agreement with the policy, risk perception, inequity, and protected values (Table 1). Questions were asked in the order shown in Table 1; no questions were asked in random order. The participants rated their interest in the Fukushima accident on a five-point scale (1 = not interested, 5 = very interested) in one question and their subjective knowledge of decontamination and the final disposal policy on a four-point scale (1 = never heard, 4 = know well) in two questions. They also rated their degree of agreement with the policy on a four-point scale (1 = disagree, 4 = agree) in the following question: “Do you agree or disagree with the policy of ‘final disposal of removed soil and other materials outside Fukushima Prefecture within 30 years after the start of interim storage (by 2045)’?” In addition, the participants were asked to answer three questions about risk perception, three questions about inequity, and two questions about protected values using a five-point scale (1 = disagree, 5 = agree) under the assumption that final disposal would occur in their neighborhood. The questions about risk perception were based on Slovic (1987) and Yokoyama et al. (2021), and those about inequity and protected values were based on Yokoyama et al. (2021) and Ohnuma et al. (2015). The questionnaire and reliability coefficients (Cronbach’s alpha) for risk perception, inequity, and protected values are shown in Table 1. Values of Cronbach’s alpha ranged from 0.86 to 0.95. The total scores of each factor were included in our analysis. The question on subjective knowledge of the final disposal policy was also used by Takada et al. (2022).

**Table 1:** Questionnaire and reliability coefficients

Factors	Questions	α^a^, Kanto region	α^a^, Kansai region
Interest in the accident	Are you interested or concerned about the Great East Japan Earthquake and Fukushima Daiichi nuclear power station accident and its recovery?	–	–
Subjective knowledge of decontamination	Did you know that after the Fukushima accident, “decontamination” was implemented to remove radioactive materials from the environment, mainly in Fukushima Prefecture?	–	–
Subjective knowledge of the final disposal policy	Did you know that according to the law the “removed soil and other materials will be finally disposed of outside Fukushima Prefecture within 30 years after the start of interim storage (by 2045)”?	–	–
Agreement with the final disposal policy	Do you agree or disagree with the policy of “final disposal of removed soil and other materials outside Fukushima Prefecture within 30 years after the start of interim storage (by 2045)”?	–	–
Risk perception	1. Final disposal site affects the health of nearby residents. 2. Final disposal site affects the surrounding environment. 3. Final disposal site affects children and the next generation.	0.95	0.94
Inequity	1. Questioning why my own rather than another neighborhood is chosen to host a final disposal site. 2. Feeling that it is unfair to have to accept hosting a final disposal site in my own neighborhood. 3. Feeling that it is unfair to accept that my own neighborhood is the only one chosen to host a final disposal site.	0.89	0.86
Protected values	1. There is no reason that having a final disposal site in my neighborhood can be acceptable. 2. There is no reason that having a final disposal site in my neighborhood can be tolerable.	0.94	0.93

^a^Cronbach’s alpha

Shirai et al. (2023) found that factors affecting the acceptability of the final disposal of contaminated soil varied with the importance of moral norms. Therefore, we expected answers to the questions in Table 1 to vary with different characteristics of moral norms. We also examined the differences according to moral foundations (Graham et al. 2011; Murayama and Miura 2019) by asking three questions for each of the five moral foundations: harm/care, fairness/reciprocity, ingroup/loyalty, authority/respect, and purity/sanctity. These questions are the same as those used in Shirai et al. (2023). After responding to the questions in Table 1, the respondents were asked to rate on a five-point scale the degree to which the question is relevant to them when determining whether an action is right or wrong (1 = not at all relevant, 5 = very relevant); for example, the question “whether or not someone was mentally injured” was used to assess the relevance of the moral foundation of harm/care. Values of Cronbach’s alpha for the five moral foundations ranged from 0.63 to 0.88 (Online Resource 2). The total scores of each moral foundation were included in our analysis. Questions on moral foundations are shown in Online Resource 2.

At the end of the questionnaire, participants could choose to share their comments and opinions on the final disposal policy, which stipulates that “removed soil and other materials will be finally disposed of outside Fukushima Prefecture within 30 years after the start of interim storage (by 2045).” Of the 2000 participants, 1524 provided responses to this section. After excluding unintelligible responses and statements of “I have no opinion,” from the provided responses, 975 valid responses were included in the text analysis. All surveys were conducted in Japanese. Questionnaires are available from the corresponding author upon request. Data from each participant are in Online Resource 3.

### Analysis

Data from both regions follow non-normal distributions (Shapiro–Wilk test, *p* < 0.05). Therefore, the Mann–Whitney U test was used to examine differences between regions, and the Spearman’s rank correlation coefficient was used to examine relationships between factors. To determine differences in the correlations between agreement with the final disposal policy and different factors, the data were first grouped according to respondents’ answers to the moral foundation questions and then the analysis above was applied to the grouped data. Scores from each moral foundation question were converted to z-scores, and a hierarchical cluster analysis was performed using the Ward method and Euclidean squared distance with five z-score totals for the three questions of each moral foundation. The number of clusters was determined using dendrograms, taking into account interpretability. Analysis was performed using R software 4.2.2 (R Core Team 2022). A probability (*p*) value of 0.05 was used as the threshold of statistical significance.

Quantitative text analysis was used to analyze the comments and opinions on the final disposal policy which enables the objective extraction of typical and representative information from large volumes of qualitative information such as comments and opinions (Higuchi 2014). Words for analysis were extracted using the text data analysis tool KH Corder (Higuchi 2016, 2017). The text analysis was conducted in Japanese and translated into English.

## Results

### Agreement with the Final Disposal Policy and Related Factors

The percentage of respondents who knew about (sum of “4 = know well” and “3 = know”) the implementation of environmental decontamination after the Fukushima accident was 66% in Kanto region and 56% in Kansai region. Thirty-three percent of the respondents in Kanto region and 22% in Kansai region knew about (sum of “4 = know well” and “3 = know”) the final disposal policy. The percentage of respondents who agreed with the policy (sum of “4 = agree” and “3 = somewhat agree”) was 65% in Kanto region and 61% in Kansai region. The results of the different factors for the two regions are shown in Table 2. Interest in the Fukushima accident, knowledge of decontamination and the final disposal policy, and agreement with the policy were significantly higher in Kanto region than in Kansai region. There was no statistically significant difference between regions in terms of risk perception, inequity, and protected values.

**Table 2:** Arithmetic mean (average), standard deviation (SD), probability (*p*) value, and effect size for different factors and regions

Factor	Region	Average	SD	*p* value	Effect size, r
Interest in the accident	Kanto	3.44	1.17	<0.001	0.10
	Kansai	3.23	1.15		
Subjective knowledge of decontamination	Kanto	2.69	0.90	<0.001	0.12
	Kansai	2.50	0.86		
Subjective knowledge of the final disposal policy	Kanto	1.98	0.97	<0.001	0.11
	Kansai	1.75	0.86		
Agreement with the final disposal policy	Kanto	2.70	0.83	0.03	0.05
	Kansai	2.64	0.77		
Risk perception	Kanto	2.72	0.74	0.86	0.004
	Kansai	2.73	0.69		
Inequity	Kanto	2.52	0.74	0.87	0.004
	Kansai	2.54	0.66		
Protected values	Kanto	1.51	0.55	0.49	0.02
	Kansai	1.53	0.51		

Correlation coefficients were examined to confirm relationships between factors (Table 3). In both regions, agreement with the final disposal policy was significantly negatively correlated with risk perception, inequity, and protected values, and significantly positively correlated with interest and knowledge of decontamination and the policy. No significant regional differences were found.

**Table 3:** Correlation coefficients between factors

	Factor 1	Factor 2	Factor 3	Factor 4	Factor 5	Factor 6	Factor 7
Factor 1. Interest in the accident	–	0.50**	0.37**	0.27**	0.24**	0.11**	*−0.01*
Factor 2. Subjective knowledge of decontamination	0.56**	–	0.51**	0.24**	0.16**	0.07*	−0.07*
Factor 3. Subjective knowledge of the final disposal policy	0.43**	0.57**	–	0.19**	*0.06*	*0.06*	*0.04*
Factor 4. Agreement with the final disposal policy	0.25**	0.27**	0.19**	–	−0.17**	−0.25**	−0.35**
Factor 5. Risk perception	0.22**	0.17**	0.01	−0.12**	–	0.68**	0.51**
Factor 6. Inequity	0.10*	0.08*	−0.03	−0.19**	0.69**	–	0.69**
Factor 7. Protected values	−0.01	−0.04	−0.01	−0.29**	0.53**	0.69**	–

Numbers in italics are Spearman’s rank correlation coefficients for Kanto region. Non-italicized numbers are Spearman’s rank correlation coefficients for Kansai region

* indicates statistical significance at the 95% confidence level (*p* < 0.05); ** indicates statistical significance at the 99% confidence level (*p* < 0.01)

### Differences According to the Importance of Moral Norms

A hierarchical cluster analysis using the z-score totals of the five moral foundations classified respondents into two clusters in each region. Cluster 1 was the group with high scores on all five moral foundations; this was considered as the group with people who value moral norms in their decisions (hereafter MH group). Cluster 2 was the group with relatively low scores on all five foundations; this was considered as the group with people who place little importance on moral norms in their decisions (hereafter ML group). The mean scores of the five moral foundations for each group are shown in Online Resource 4. In Kanto region, there were 663 respondents in the MH group and 337 respondents in the ML group. In Kansai region, there were 337 and 663 respondents in the MH and ML groups, respectively. In both regions, average scores for six of the seven factors were significantly higher in the MH group than in the ML group. For agreement with the final disposal policy (hereafter policy approval), the score of the MH group was significantly higher than that of the ML group in Kanto region; in Kansai region, there was no statistically significant difference between MH and ML scores for policy approval (Online Resource 5).

Correlation coefficients between policy approval and other factors were examined for the different groups (Table 4). The correlation coefficients between policy approval and risk perception, inequality, and protected values were significantly negative for both groups except for the ML group in Kanto region. Fisher’s Z-transformation was used to test if the correlation coefficients of the MH and ML groups from the same region were statistically significantly different; *p-*values indicate that the correlation coefficients between policy approval and risk perception for MH groups were significantly smaller than those for ML groups. The correlation coefficients between policy approval and interest in the accident and knowledge of decontamination and the policy were statistically significant for both groups; coefficients for ML groups were significantly larger than those for MH groups, with the exception of a statistically insignificant difference between the two groups in Kansai region in terms of their knowledge of the policy. The correlation coefficients between the seven factors for both MH and ML groups and Kansai and Kanto regions are shown in Online Resource 6.

**Table 4:** Correlation coefficients between agreement with the final disposal policy and other factors in Kanto and Kansai regions for the MH group (people who value moral norms in their decisions) and the ML group (people who place little importance on moral norms in their decisions)

Correlation coefficient between agreement with the final disposal policy and different factors	Kanto region			Kansai region		
	MH	ML	*p*-value of Fisher’s Z-transformation test	MH	ML	*p*-value of Fisher’s Z-transformation test
Interest in the accident	0.19**	0.32**	0.04	0.17*	0.30**	0.04
Subjective knowledge of decontamination	0.20**	0.34**	0.02	0.14*	0.28**	0.03
Subjective knowledge of the final disposal policy	0.13**	0.30**	<0.01	0.16**	0.20**	0.54
Risk perception	−0.20**	−0.06	0.03	−0.35**	−0.12**	<0.01
Inequity	−0.26**	−0.14**	0.06	−0.34**	−0.24**	0.10
Protected values	−0.32**	−0.23**	0.15	−0.42**	−0.34**	0.16

Values in the MH and ML columns are Spearman’s rank correlation coefficients

* indicates statistical significance at the 95% confidence level (*p* < 0.05); ** indicates statistical significance at the 99% confidence level (*p* < 0.01)

### Text Analysis of Comments and Opinions on the Policy

**Fig. 1:**
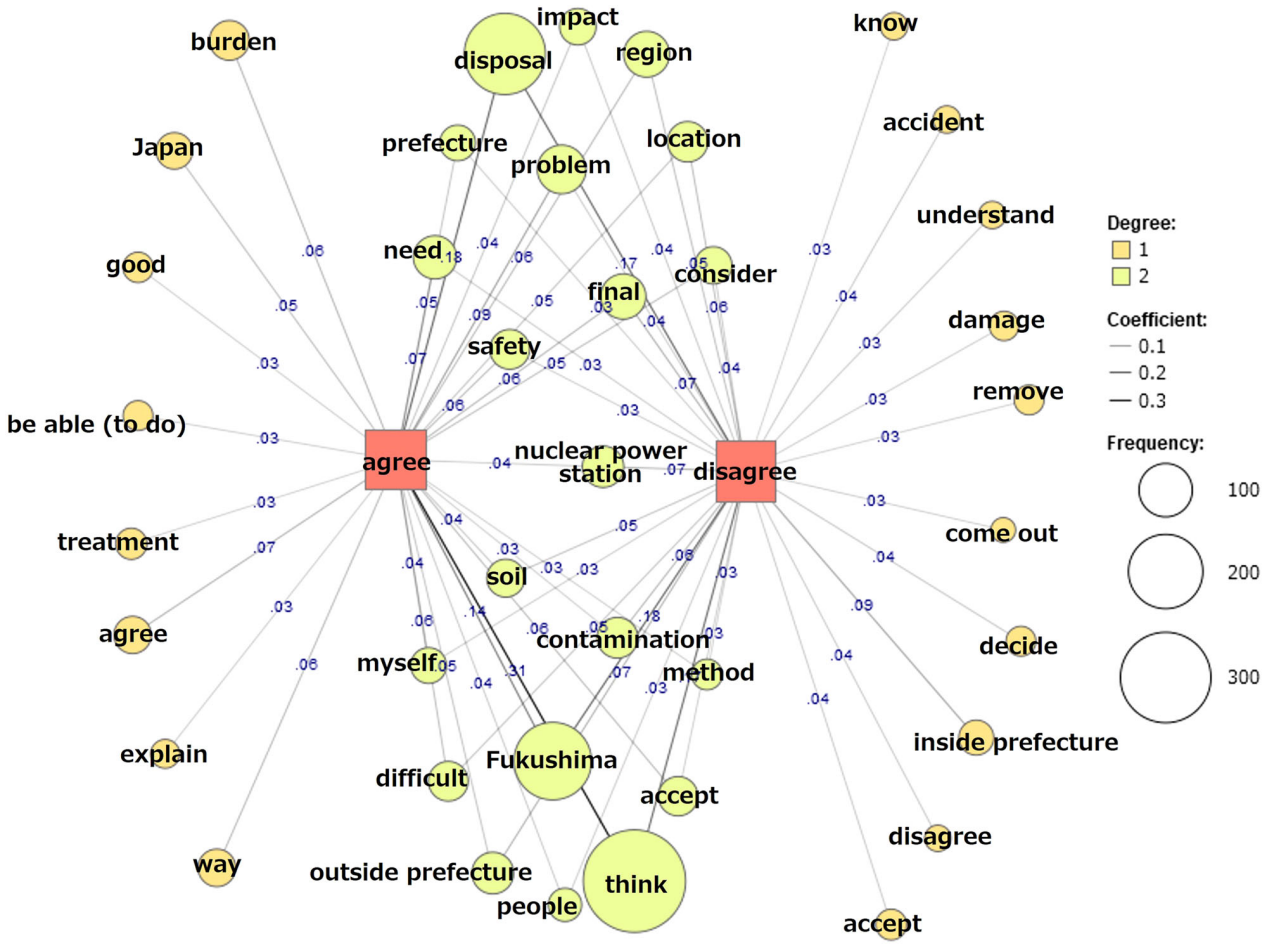
Co-occurrence network of frequently occurring words in comments or opinions from the participants on the final disposal policy. The external variables were “agree (agree and somewhat agree)” and “disagree (disagree and somewhat disagree)”

There were 975 comments and opinions on the final disposal policy. In Kanto region, 335 respondents agreed or somewhat agreed with the policy and 181 respondents disagreed or somewhat disagreed. In Kansai region, 279 respondents agreed or somewhat agreed with the policy and 180 disagreed or somewhat disagreed. We analyzed 1202 sentences and generated a co-occurrence network (Fig. 1) with the 60 most frequently used words, which have each appeared at least 15 times (Online Resource 7). The external variables were “agree (agree and somewhat agree)” and “disagree (disagree and somewhat disagree).” Words connected to both external variables were common words that were used independently of opinion, while words connected only to either agree or disagree were words found in specific opinions or comments.

Frequently occurring words that were specific to respondents who agreed with the policy were “Japan,” “burden,” and “way (the negative form of ‘no way’ was predominant in the context).” Examples of comments and opinions are as follows:

*I agree. I think Japan as a whole needs to think about this issue instead of putting the burden on Fukushima Prefecture alone. (Kanto region)*

*I think this is a problem that should be solved in Fukushima because it originally happened in Fukushima, but solving it only in Fukushima is impossible from a practical point of view, so we have no way but to bear the burden as Japanese citizens. (Kansai region)*

*The fact is that we have to dispose of it somewhere, and there is no way around it. (Kansai region)*

Frequently occurring words that were specific to respondents who disagreed with the policy were “inside Prefecture,” “accept (the negative form of ‘cannot accept’ was predominant in the context)” and “know (the negative form of ‘do not know’ was predominant in the context).” Examples of comments and opinions are as follows:

*I did not know about such a policy. (Kansai region)*

*Final disposal should be conducted inside Fukushima Prefecture because residents have benefited from the nuclear power station until now. (Kanto region)*

*In any case, lack of explanation and top-down decisions by the government are unacceptable. (Kanto region)*

*I have vague anxiety. The reasons are not convincing, and I cannot accept it. (Kanto region)*

All comments and opinions are available in Online Resource 3.

## Discussion

### Factors Affecting Approval of the Final Disposal Policy

In the present study, 33% of respondents in Kanto region and 22% in Kansai region knew about the final disposal policy. In the 2019 survey conducted by Takada et al. (2022), approximately 20% of respondents from all regions of Japan except Fukushima Prefecture knew about the policy, indicating that the level of awareness has not changed significantly over three years. Our results show that more than 60% of the respondents agreed with the policy, which was higher than the percentage of respondents who knew about the policy. This means that even if the respondents did not know about the policy before participating in the survey, they would agree with it based on the information presented in the survey. Policy approval was positively correlated with interest in the accident and knowledge of decontamination and the policy, and negatively correlated with risk perception, inequity, and protected values. Previous studies have shown that risk perception, inequity, and protected values have negative effects on the acceptance of NIMBY facilities after the selection of a candidate site (Slovic 1987; Slovic et al. 1991; Baron and Spranca 1997; Sjöberg 2004; Krütli et al. 2012; Yokoyama et al. 2021; Takada et al. 2022). Our results show that these factors have similar effects on the public’s attitudes toward the final disposal policy. Our results show strong positive correlations between risk perception, inequality, and protected values; this is in agreement with a previous study on acceptance of the recycling of contaminated soil, which reported positive correlations between inequity and factors disturbing acceptance such as risk perception and protected values (Yokoyama et al. 2021). Interest in the accident and knowledge of decontamination and the final disposal policy were higher in Kanto region than in Kansai region, probably because of the proximity of Kanto region to Fukushima. Policy approval was also slightly higher in Kanto region; this may reflect the higher levels of knowledge and interest in Kanto region, which positively impact approval level. There were no regional differences in perceived risk, inequity, or protected values, providing insufficient evidence to conclude that these factors are influenced by distance from Fukushima or parties’ sense of involvement in the accident.

In both regions, respondents were divided into the MH group, which places more importance on moral norms in judgment, and the ML group, which places less importance on moral norms in judgment. Correlations between policy approval and different factors varied between groups; risk perception had a larger effect on policy approval in MH groups than in ML groups. These results are consistent with those of Shirai et al. (2023) who reported that risk perception negatively impacted acceptance of the final disposal but the impact was smaller in the group with low moral foundation score. We found that knowledge of decontamination and interest in the accident positively impacted policy approval and the impact were greater for ML groups than for MH groups. This indicates that simply having interest in and knowledge of Fukushima and the final disposal policy were effective in improving ML group’s approval of the policy but had a small effect on the MH group.

In the comments and opinions on the final disposal policy, the words “Japan,” “burden,” and “no way” were frequently used by respondents who agreed with the final disposal policy; this suggests that people who approved of the policy emphasized the concept of burden sharing. In line with our findings, previous studies have also reported that burden sharing was associated with acceptance of the final disposal and recycling of contaminated soil of Fukushima (Yokoyama et al. 2021, Takada et al. 2022). The words that were frequently used by respondents who disagreed with the final disposal policy indicate that these respondents were not informed about the policy and that they considered the policy as a top-down decision, which they disagreed with. This indicates a lack of information disclosure and transparency, which are important elements of procedural fairness. In line with our findings, procedural fairness has been found to be associated with acceptance of NIMBY facilities (Kuhn and Ballard 1998; Besley 2010; Ohtomo et al. 2014; Takada et al. 2022) and is an important concern especially for people who oppose these projects (Hirose 2007). Comments frequently expressed respondents’ disapproval of disposing of the contaminated soil outside Fukushima Prefecture. This suggests that the rationale behind the Cabinet decision of disposal outside Fukushima Prefecture—“as a result of a comprehensive judgment based on the fact that residents in Fukushima Prefecture are already bearing an excessive burden”—was insufficient to gain the support of those who oppose the policy.

### Implications

The present study indicates that, as of 2022, many members of the public support the policy of the final disposal of contaminated soil outside Fukushima Prefecture, although public knowledge of the policy is not widespread. Broadening the government’s appeal to all citizens in Japan about the final disposal policy may increase public support for the policy. Forming a common understanding of the implications of disposal outside Fukushima prefecture, in which other prefectures share the burden, may also have the effect of increasing support, as suggested by Yokoyama et al. (2021) and Takada et al. (2022). Factors affecting public attitudes toward the policy are consistent with findings on the acceptance of NIMBY facilities after site selection: in the hypothetical situation of hosting a final disposal site in one’s own neighborhood, residents with a perception of a high level of risk, a sense of inequity, and protected values were more likely to be reluctant to support the policy. People who had negative attitudes toward the policy considered that there were problems with information disclosure and transparency, which are important elements of procedural fairness, and that there were insufficient reasons and explanations for moving the contaminated soils out of Fukushima Prefecture. To gain the support of these people, sufficient attention must be paid to procedural fairness. Furthermore, from the perspective of reducing the sense of inequity, disposal at multiple locations could be considered. The Ministry of Environment’s efforts to disseminate information and inform the public about recycling and disposal outside Fukushima Prefecture (Ministry of the Environment 2023d) may be effective in gaining support from some people. For others, however, especially those who value moral norms in their decision making, information dissemination alone is insufficient to obtain their support for the policy. An honest and detailed explanation of the risks and special attention to procedural fairness would also be essential.

### Limitations

The limitations of the present study are as follows: we used a web-based survey and received responses from 2000 respondents. The respondents were registered with the online survey company, and it has been indicated that they may differ from the general population in terms of income, education, and other factors (e.g. Fleming and Bowden 2009). Therefore, our results need to be verified by other methods, such as a mail survey. It should also be noted that the survey was limited to Kanto and Kansai regions. As a result, the results do not reflect the attitudes of the entire Japanese public. Our results indicate that residents in Kanto region were more interested and subjectively knowledgeable than those in Kansai region, and were more supportive of the disposal policy. Considering that there are regions of Japan that are at greater distances than Kansai region from Fukushima and the area that benefits from TEPCO’s services, national average levels of subjective knowledge, interest, and approval of the final disposal policy could be lower than the results from the present study. These results were obtained in 2022 when public knowledge of the final disposal policy was not high. There may be substantial future changes in public attitudes depending on how the government implements the policy (e.g., the process for site selection). On the basis of respondents’ comments and opinions, we were able to identify differences between viewpoints; reasons behind respondents’ opinions need to be determined by future interviews. In this study, we examined the relationship between agreement with the final disposal policy and factors such as interest in and knowledge of decontamination and the policy, risk perception, inequality, and protected values. Trust in government, as noted in the previous study on the removed contaminated soil (Shirai et al. 2023), and communication and scientific literacy of the public may also be important factors. To examine the influence of these factors on people’s agreement with the policy, additional studies are needed.

## Conclusions

This study aimed to identify factors that are important for building consensus over the final disposal of contaminated soil from the Fukushima accident. We examined the attitudes of the general public toward the policy of the final disposal through a web-based survey. Respondents were from Kanto region, the beneficiary area of the Fukushima nuclear power station, and a control region. Study results provide important indications that increasing public awareness of current efforts toward final disposal creates some support for the policy, but is insufficient for obtaining nationwide understanding of the issue. Procedural fairness needs to be addressed, including explanations of risk, information disclosure, and transparency. Procedural fairness is essential, especially for people who have a negative attitude toward the policy.

Our findings have important implications in terms of focusing public discussion and improving public understanding of the final disposal policy prior to the selection of candidate site(s). In addition, they may provide useful information for addressing the issue of the disposal of large quantities of radioactively contaminated materials from a large-scale nuclear accident.

### Supplementary information

Supplementary Information

Resource 2-6
