# Supplementary material for: Public Attitudes toward the Final Disposal of Radioactively Contaminated Soil Resulting from the Fukushima Daiichi Nuclear Power Station Accident
Source: Environ Manage. 2024 Feb 2;73(5):962–72. doi: 10.1007/s00267-024-01938-w (PMC11023960; doi:10.1007/s00267-024-01938-w)
Supplement: Supplementary file 1 — Supplementary Information [file 267_2024_1938_MOESM1_ESM.pdf]

Supplementary Information

Article title: Public attitudes toward the final disposal of radioactively contaminated soil resulting from the Fukushima Daiichi nuclear power station accident

Journal name: Environmental Management

Author names, and affiliation and e-mail address of the corresponding author:
Momo Takada1*, Michio Murakami2, Susumu Ohnuma3, Yukihide Shibata3, Tetsuo Yasutaka1
1 Institute for Geo-Resources and Environment, National Institute of Advanced Industrial Science and Technology, Tsukuba, Ibaraki, Japan
2 Center for Infectious Disease Education and Research, Osaka University, Suita, Osaka, Japan
3 Department of Behavioral Science, Graduate School of Letters, Hokkaido University, Sapporo, Japan
* Corresponding author: momo.takada@aist.go.jp

概要説明

◇放射性セシウムによる環境汚染
2011年3月に発生した東日本大震災による地震と津波の影響により、東京電力福島第一原子力発電所事故(以下、「原発事故」という)が発生しました。この原発事故により、放射性物質が大気中に放出され、福島県を中心に、東北地方、関東地方の一部の地表や建物、樹木、土壌等を汚染しました。

◇環境除染
事故由来の放射性物質による汚染が、人の健康又は生活環境に及ぼす影響を低減するため、福島県を中心に環境中から放射性物質を取り除く「除染」が2012年から約5年かけて実施され、福島県内を中心に約1300万㎥(東京ドーム約11杯分)の除去土壌(除去された汚染土壌は、政策上「除去土壌」と呼ばれていますので、この説明でもこの用語を使います)が発生しました。
現在までに除去土壌の多くは、福島第一原子力発電所の隣接地に建造された中間貯蔵施設に運び込まれています。2020年度までに除染に関係する費用は約5兆円かかっています。

◇現在の状況と今後
中間貯蔵施設に保管された除去土壌や廃棄物は、中間貯蔵開始後30年以内(2045年まで)に、福島県外で最終処分を完了することが法律で決まっています。「福島県外」での最終処分は、「福島県においては住民が既に過重な負担を負っており、総合的に判断した結果」であり(第187回国会衆議院環境委員会議録第5号)、2011年に閣議決定されました。
最終処分については、中間貯蔵開始後30年以内に福島県外で処分されること以外、決まっていることはなく、最終処分地の数(1箇所なのか、複数箇所なのか)やどのような手続きで決めるのか、も決まっていません。

本アンケートでは、1300万㎥の除去土壌を対象として、2045年までの福島県外での最終処分について、皆様のご意見をお聞きするものです。

Online Resource 1. Preliminary explanation of the final disposal that respondents had to read before accessing the questionnaire.

Preliminary explanation

◆ Environmental Contamination by Radiocesium
The earthquake and tsunami caused by the Great East Japan Earthquake in March 2011 resulted in the accident at TEPCO's Fukushima Daiichi Nuclear Power Station (hereafter "nuclear accident"). As a result of the nuclear accident, radioactive materials were released into the atmosphere, contaminating the ground surface, buildings, trees, soil, and other elements in Fukushima Prefecture and other parts of the Tohoku and Kanto regions.

◆ Environmental Decontamination
In order to reduce the impact of contamination by radioactive materials from the accident on human health and the living environment, "decontamination", i.e. the removal of radioactive materials from the environment, was carried out mainly in Fukushima Prefecture over a period of about five years starting in 2012. About 13 million m^3 (about 11 times the size of the Tokyo Dome) of soil was removed mainly in Fukushima Prefecture (contaminated soil removed from the environment is referred to as "removed soil" in the policy so we will also use this terminology in this explanation).
Most of the removed soil has been transported to an interim storage facility adjacent to Fukushima Daiichi Nuclear Power Station. The cost of decontamination through FY2020 is approximately 5 trillion yen.

◆ Situation and Future
The law requires the removed soil stored in the interim storage facility to be transported outside Fukushima Prefecture for final disposal within 30 years after the start of interim storage (by 2045). The decision to implement final disposal outside Fukushima Prefecture is "a result of a comprehensive judgment based on the fact that residents in Fukushima Prefecture are already bearing an excessive burden" (187th Diet Session, House of Representatives, Committee on the Environment, Minutes No. 5), which was approved by the Cabinet in 2011.
Nothing has been decided about final disposal except that the removed soil will be disposed of outside Fukushima Prefecture within 30 years after the start of interim storage, and the number of final disposal sites (one or more sites), and the decision procedures have still to be determined.

In this survey, we are asking for your opinion on the final disposal of the 13 million m^3 of removed soil outside Fukushima Prefecture by 2045.
